# Accuracy evaluation of CAD/CAM generated splints in orthognathic surgery: a cadaveric study

**DOI:** 10.1186/s13005-015-0082-9

**Published:** 2015-07-25

**Authors:** Thomas Schouman, Philippe Rouch, Benoît Imholz, Jean Fasel, Delphine Courvoisier, Paolo Scolozzi

**Affiliations:** Department of Surgery, Service of Maxillofacial and Oral Surgery, University Hospital and Faculty of Medicine, Geneva, Switzerland; Hôpital Pitié-Salpêtrière, Service de Chirurgie Maxillofaciale et Stomatologie, UPMC Université Paris, Paris, France; Arts et Métiers ParisTech, LBM, 151, Boulevard de l’hôpital, Paris, France; Department of Anatomy, Faculty of Medicine - University of Geneva, Geneva, Switzerland; CRC & Division of Clinical Epidemiology, Department of Health and Community Medicine, University of Geneva & University Hospitals of Geneva, Geneva, Switzerland

**Keywords:** Orthognathic surgery, Computer-assisted surgery, CAD/CAM splints

## Abstract

**Introduction:**

To evaluate the accuracy of CAD/CAM generated splints in orthognathic surgery by comparing planned versus actual post-operative 3D images.

**Methods:**

Specific planning software (SimPlant^®^ OMS Standalone 14.0) was used to perform a 3D virtual Le Fort I osteotomy in 10 fresh human cadaver heads. Stereolithographic splints were then generated and used during the surgical procedure to reposition the maxilla according to the planned position. Pre-operative planned and postoperative 3D CT scan images were fused and imported to dedicated software (MATLAB^®)^ 7.11.) for calculating the translational and rotational (pitch, roll and yaw) differences between the two 3D images. Geometrical accuracy was estimated using the Root Mean Square Deviations (RMSD) and lower and upper limits of accuracy were computed using the Bland & Altman method, with 95 % confidence intervals around the limits. The accuracy cutoff was set at +/− 2 mm for translational and ≤ 4° for rotational measurements.

**Results:**

Overall accuracy between the two 3D images was within the accuracy cutoff for all values except for the antero-posterior positioning of the maxilla (2.17 mm). The translational and rotational differences due to the splint were all within the accuracy cutoff. However, the width of the limits of agreement (range between lower and upper limits) showed that rotational differences could be particularly large.

**Conclusion:**

This study demonstrated that maxillary repositioning can be accurately approximated and thus predicted by specific computational planning and CAD/CAM generated splints in orthognathic surgery. Further study should focus on the risk factors for inaccurate prediction.

## Introduction

Treatment planning in orthognathic surgery is based on a combination of clinical, radiological and plaster casts analyses. These analyses allow for a simulation of the ideal repositioning of the skeletal pieces of the facial skeleton that should be reproduced during the surgery as closely as possible to the simulation. Usually, surgical intermediate and final occlusal acrylic splints made on plaster models mounted on a semi-adjustable articulator, after facebow transfer, are used to reproduce the planning during surgery. This method has numerous and inherent sources of non-controllable errors. The succession of manipulations and the multiple stakeholders implied make this hand-made planning reliability questionable [1–5]. The accuracy of this method cannot be estimated by making preoperative and postoperative clinico-radiological comparisons. Such comparisons only provide a global approximation of the whole process and do not allow differentiating the errors due to patient registration, model surgery, surgical technique, and method of comparison itself [1–5].

New methods of 3D virtual planning integrating fully digitized clinical and radiological data are now fully efficient and surgical wafers can also be generated from these data, without the need for additional human interference [6–16]. These methods presumably offer the highest accuracy of treatment planning, but the overall accuracy of the planning and of its surgical reproducibility has not yet been quantified. The aim of the present cadaveric study was to evaluate the accuracy of CAD/CAM generated splints in orthognathic surgery.

## Materials and methods

To address the research purpose, the authors designed and implemented an experimental study using 10 fresh human cadaver heads for evaluating the accuracy of CAD/CAM stereolithographic surgical splints. The specimens were obtained from the Division of Anatomy of our University after the required authorization was given by the legally responsible person and the study was approved by our hospital ethical board (Study 10–274).

### Technical procedure

#### 3D coordinate system and reference points

**Fig. 1:**
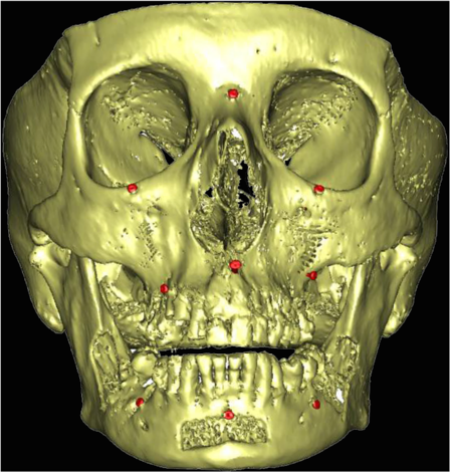
Frontal 3D CT scan image view showing the nine cortical bone screws (*in red*) placed as references to define a three-dimensional coordinate system (3 in the skull, 3 in the maxilla and 3 in the mandible)

Nine 1.5 mm diameter titanium monocortical screws (*Synthes®-CH 4436 Oberdorf, Switzerland*) were inserted in each human cadaver head as follows: a) three screws within the skull (one within the nasion and one within the left and right infraorbital rim). These screws were used as points of reference (fiducial markers) to define a three-dimensional coordinate system; b) three screws within the maxilla (one within the maxillary midline underneath the anterior nasal spine and one above the roots of the left and right upper first molars); c) three screws in the mandible (one within the mental midline underneath the lower central incisors and one underneath the roots of the lower left and right first molars) (Fig. 1).

#### Image acquisition

Preoperative imaging was performed with a 64-slice CT scanner (Siemens Sensation 64; Germany: 120 kV; 240 mAS; 2 9 32 detectors; increment, 0.7 mm; collimation, 64 9 0.6; slice thickness, 1 mm; matrix, 512 9 512 pixels; gantry tilt, 0°).

#### Pre-operative computational image analysis

CT scan images in DICOM (Digital Imaging and Communications in Medicine) format were processed using SimPlant OMS Standalone 14.0 software (SIMPLANT Business Unit, Technologielaan 15, 3001 Leuven, Belgium www.materialisedental.com). The dental casts obtained from alginate dental impressions were scanned using a high-resolution 3D optical scanner (Dental 3D Scanner D-200™, http://www.3shape.com). The dental scan images were then imported to the software and superimposed on the CT scan images by means of a semi-automated 3D surface registration (*Iterative Closest Point registration*). In two partially edentulous cases, the missing teeth were replaced by a prosthesis fixed with bone screws to obtain a stable occlusal platform.

#### 3D virtual surgical planning

**Fig. 2:**
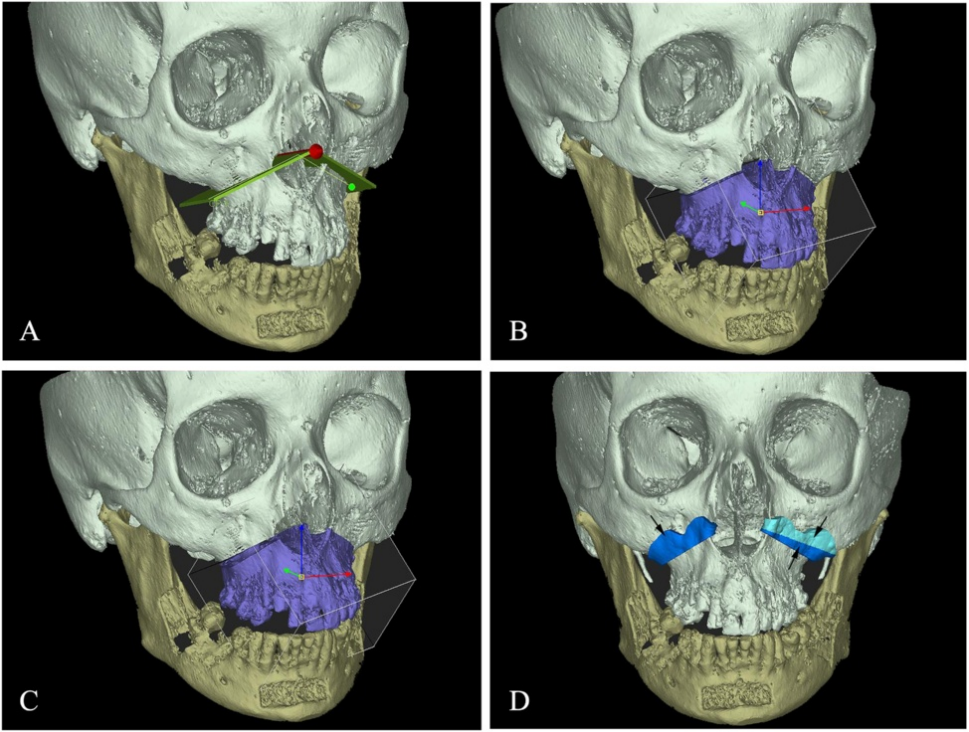
**a** Generation of the plane for the virtual maxillary’s osteotomy (**b**) Segmentation of 3-D bone segments corresponding to the Le Fort I osteotomy (**c**) Repositioning of the maxillary osteotomized segment according to the planned movements (**d**) Generation of specific maxillary cutting guides to reproduce the planned virtual Le Fort I-type osteotomy (*arrows*)

The 3D-coordinate system was integrated into the 3D-model with X, Y, and Z axes corresponding respectively to the medio-lateral axis, antero-posterior axis and infero-superior axis. The plane for the virtual Le Fort I osteotomy was first generated (Fig. 2a) and then the 3D maxillary bone was segmented (Fig. 2b). The maxillary digitally osteotomized segment was repositioned to simulate the planned and arbitrarily chosen movements as follows (Fig. 2c):

- *Maxillary advancement:* 5 mm
- *Superior maxillary repositioning on the left side:* 4 mm at the pterygo-maxillary and 3 mm at the naso-maxillary buttresses
- *Inferior maxillary repositioning on the right first side:* 3 mm at the pterygo-maxillary buttress.

The intermediate splint was thus designed according to the new maxillary position as well as specific maxillary cutting guides to reproduce the planned virtual Le Fort I-type osteotomy (Fig. 2d).

#### CAD/CAM surgical splint

Stereolithographic splints were generated based upon the treatment planning as follows:

The .STL file (*Standard Tesselation Language or Stereolithography format*) of the new maxillo-mandibular relationship was converted into a layer-by-layer contour model. A new part-specific file was generated to be run on the stereolithographic machine. The splints were then fabricated (3D printing) by using Triad®TranSheet^TM^ material from DentSply (http://www.dentsply.com/en).

**Fig. 3:**
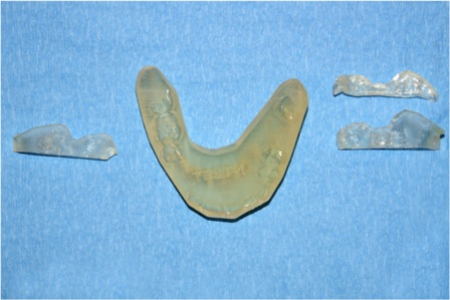
The final stereolithographic splint and cutting guides

Data were used then to create the specific surgical cutting guides (Fig. 3).

#### Surgical procedure

**Fig. 4:**
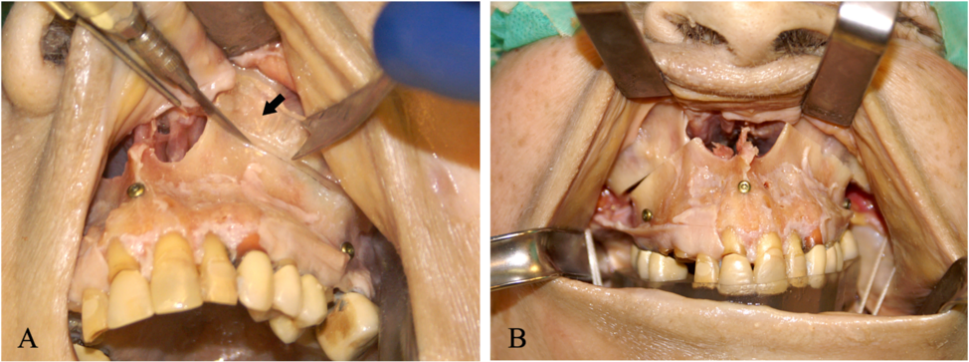
**a** Complete Le Fort I-type osteotomy performed with a reciprocating saw by using specific maxillary cutting guides (*black arrow*) (**b**) Maxillo-mandibular repositioning according to the planned movements with the intermediate stereolithographic occlusal splint

The surgical procedure has been performed by the same surgeon (T.S). A complete Le Fort I-type osteotomy was performed with a reciprocating saw by using specific maxillary cutting guides. The maxilla was then down fractured. The bone resection on the left side of the maxilla needed for asymmetrical intrusion was carried out according to the measurements made at the internal reference points following the virtual surgical planning. To secure the maxilla in its new position, a maxillo-mandibular fixation (MMF) with the intermediate stereolithographic occlusal splint was performed using peri-zygomatic non-metallic ligatures posteriorly and a ligature between a screw within the nasion and a screw within the symphysis anteriorly (Fig. 4a–c).

#### Post-operative computational image analysis

**Fig. 5:**
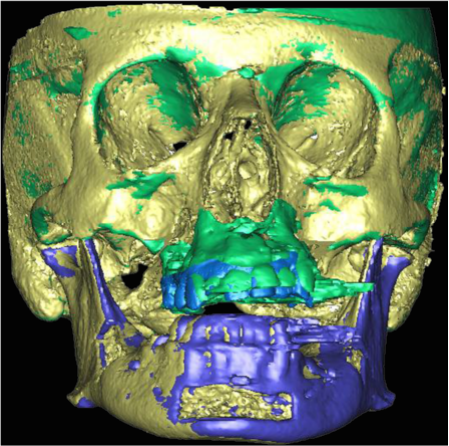
Planned pre- and post-operative 3D CT scan images fused by means of an automated surface matching method by using the skull, which was not repositioned by the surgery, as reference for registration

Post-operative CT-scans with the intermediate splint in place were taken using the pre-operative protocol. Planned pre- and post-operative 3D CT scan images were fused by means of an automated surface matching method by using the skull, which was not repositioned by the surgery, as reference for registration (Fig. 5). The differences between the two images were calculated by using MATLAB® 7.11 (R2010b) software (MathWorks 92190 Meudon France http://fr.mathworks.com/products/matlab/ ) as follows:

1. The three following 3D anatomical regions were defined for the final evaluation: a) skull, b) maxilla, and c) mandible.
2. The position of three fixed points (screws) within the 3D-coordinate system of the CT scan was determined on the planned pre- (Pt_plan1_ Pt_plan2_ Pt_plan3_)^*1^ and post-operative (Pt_ppop1_ Pt_ppop2_ Pt_ppop3_ ) ^*2^ 3D CT scan images. ^*1^_*plan:*_= planned;^*2^_*ppop*_*:post-operative*
3. A landmark (*barycenter*) “rigidly” related to the three fixed points (screws) was then calculated for each anatomical region (skull, maxilla and mandible) in the planned and post-operative images as follows:

**Table:** 

*X*_bary_ =	*X*Pt_plan1_ + *X*Pt_plan2_ + *X*Pt_plan3_	*X*_bary_ =	*X*Pt _ppop1_ + *X*Pt _ppop2_ + *X*Pt _ppop3_
	3		3
*Y*_bary_ =	*Y*Pt_plan1_ + *Y*Pt_plan2_ + *Y*Pt_plan3_	*Y*_bary_ =	*Y*Pt _ppop1_ + *Y*Pt _ppop2_ + *Y*Pt _ppop3_
	3		3
*Z*_bary_ =	*Z*Pt_plan1_ + *Z*Pt_plan2_ + *Z*Pt_plan3_	*Z*_bary_ =	*Z*Pt _ppop1_ + *Z*Pt _ppop2_ + *Z*Pt _ppop3_
	3		3
  (*X*Pt_1_*X*Pt_2_*X*Pt_3_) (Y_Pt1_*Y*Pt_2_*Y*Pt_3_) (Z_Pt1_*Z*Pt_2_*Z*Pt_3_) represented the spatial coordinates of the three points (screws) within each anatomical region.
4. The pre- and post-operative barycenters (Bary_plan_ and Bary_ppop_) corresponding to the three anatomical regions (skull, maxilla and mandible), whose axis were collinear to those of the CT scan, were thus taken as references for determining the translational and rotational (pitch, roll and yaw) measurements (Fig. 6).

**Fig. 6:**
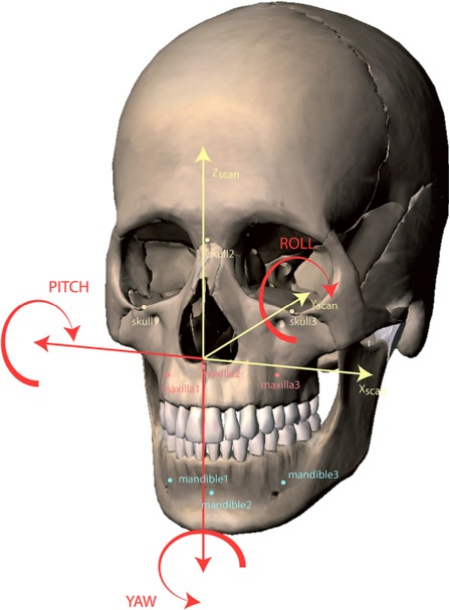
3D CT scan image view showing the rotational (pitch, roll and yaw) movements measured
5. Three vectors on the planned pre- (V_plan1_;V_plan2_;V_plan3_) and post-operative (V_ppop1_;V_ppop2_;V_ppop3_) were then determined to describe the space of the three anatomical regions (Fig. 7):

**Fig. 7:**
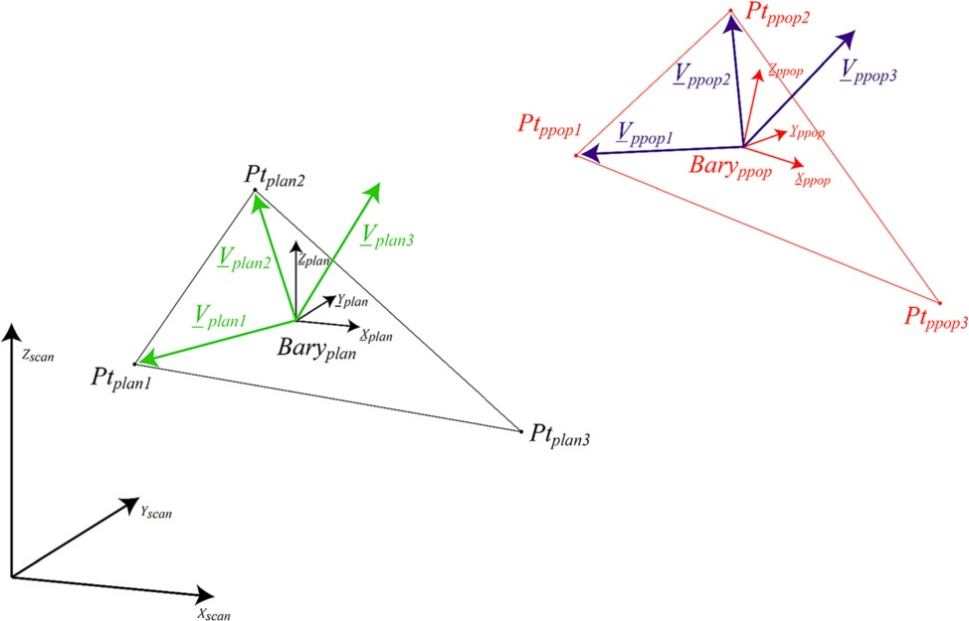
The pre- and post-operative barycenters (Bary _plan_ and Barry _ppop_) corresponding to the three anatomical regions (skull, maxilla and mandible) and the three vectors on the planned pre- (V_plan1_;V_plan2_;V_plan3_) and post-operative (V_ppop 1_;V_ppop 2_;V_ppop 3_) were then determined to describe the space of the three anatomical regions

**Table:** 

V_plan1_=	Bary_plan_ Pt_plan1_	V_ppop1_=	Bary_ppop1_ Pt_ppop1_
V_plan2_=	Bary_plan_ Pt_plan2_	V_ppop 2_=	Bary_ppop2_ Pt_ppop2_
V_plan3_=	Bary_plan_ Pt_plan3_	V_ppop 3_=	Bary_ppop3_ Pt_ppop3_
6. Finally, the measurements of the translational movements were calculated from the length of the corresponding vector connecting the pre- and post-operative barycenters (Mov = Bary_plan_Bary_ppop_) whereas the measurements of rotational (pitch, roll and yaw) movements were calculated from the following transfer matrices:
  $$ \mathrm{pitch}= \arctan \left[\frac{\mathrm{Yppop}\ z}{\mathrm{Yppop}\ y}\right] $$
  $$ \mathrm{roll}= \arctan \left[\frac{\mathrm{Yppop}\ x}{\mathrm{Yppop}\ y}\right] $$
  $$ \mathrm{yaw}= \arctan \left[\frac{\mathrm{Yppop}\ y}{\mathrm{Yppop}\ x}\right] $$
7. The difference between the positions of the screws on the pre- versus post-operative images due to the screw’s deformation related to the MMF as well as to the skull’s manipulations was also calculated for the three anatomical regions and was labeled as the *inter-points distance (IPD)*.

### Statistical analysis

Data were analyzed using R 3.1.1 statistical software (R Development Core Team, Vienna, Austria). Geometrical accuracy was estimated using Root Mean Square Deviations (RMSD = square root (1/n sum (d^2^)), which were computed for each axis on the orientation and angle differences for the mandible and the maxilla. In addition, lower and upper limits of accuracy were computed using the Bland & Altman method, with 95 % confidence intervals around the limits. The upper and lower limits *l* are given by d ± 1.96× SD, and the confidence interval around the limits are given by:

$$ l\hbox{-} t\ \sqrt{\frac{3S{D}^2}{n}} $$

where $\sqrt{\frac{3S{D}^2}{n}}$ is the standard error of the limit and t is the critical value for the t distribution (2-tailed at 0.05).

Overall accuracy as a function of each head was estimated by calculating the translational and rotational differences in the three-dimensional coordinate system (x: medio-lateral; y: antero-posterior and z: supero-inferior) between pre- and post-operative skull, maxilla and mandible and represented the imprecision related to the whole procedure (computational, manufactural and surgical). The differences determined for each cranial region accounted for the accuracy of the computational process used for determining splint accuracy. The differences determined for the mandible resulted from the accuracy of the computational process and the condylar mandibular repositioning error. Finally, the differences determined for the maxilla resulted from the accuracy of the computational process, the condylar mandibular repositioning error plus the intrinsic error related to the splint and could be calculated as follows: MxRE = Md RE + SE*. Thus, the intrinsic error due to the splint could be estimated by calculating the difference in 3D deviations between maxilla and mandible as follows:

$$ \mathrm{S}\mathrm{E}=\mathrm{MxRE}\ \hbox{-}\ \mathrm{M}\mathrm{d}\ \mathrm{R}\mathrm{E}*. $$

According to the literature, a translational difference of less than 2 mm and an orientation difference of less than 4° were considered to be good accuracy [16–18].

Finally, the RMSD of the *inter-points (IPD)* was calculated and compared to the overall RMSD.

*MxRE: Maxillary repositioning error, MdRE: Mandibular repositioning error, SRE: Splint error

## Results

The 3D translational and rotational differences measured between planned and post-operative cranial region demonstrated good accuracy of the whole imaging computational process ranging from 0.00 to 0.20 mm for translational and 0.10° to 0.67° for rotational movements. The 3D translational and rotational differences measured between planned and post-operative mandible and maxilla were found to be within the permitted accuracy cutoff except for the antero-posterior positioning of the maxilla, which was slightly beyond this limit (2.17 mm) (Table 1). However, the Bland & Altman method showed a greater variability of the extreme values with the lower limits of the translational differences exceeding 2 mm in the medio-lateral axis for both the mandible and the maxilla. Conversely, the upper limits were all beyond the admitted values except in the supero-inferior axis for the maxilla (Table 2). With respect to rotational differences, the lower limits were all within 4° for the mandible and for roll and yaw for the maxilla. The upper limits were all within 4° for the mandible and only for yaw for the maxilla.

**Table 1:** Overall accuracy (*Root Mean Square Deviation*) of 3D translational and rotational differences between the planned and post-operative images

	Translational difference		Rotational difference	
Skull	Mediolateral	0.05	Pitch	0.67
	Anteroposterior	0.17	Roll	0.31
	Superoinferior	0.20	Yaw	0.10
Mandible	Mediolateral	2.00	Pitch	1.03
	Anteroposterior	1.69	Roll	0.63
	Superoinferior	1.23	Yaw	1.09
Maxilla	Mediolateral	1.55	Pitch	3.70
	Anteroposterior	2.17	Roll	2.06
	Superoinferior	0.81	Yaw	0.93

**Table 2:** Overall accuracy (*Bland-Altman upper and lower limits*) of 3D translational and rotational differences between the planned and post-operative images

	Translational difference (95 % CI)			Rotational difference (95 % CI)		
		Lower limit	Upper limit		Lower limit	Upper limit
Skull	Mediolateral	−0.08 (−0.14 to −0.02)	0.11 (0.05 to 0.17)	Pitch	−1.52 (−2.25 to −0.79)	0.83 (0.10 to 1.56)
	Anteroposterior	−0.39 (−0.61 to −0.18)	0.29 (0.08 to 0.51)	Roll	−0.65 (−1.05 to −0.25)	0.64 (0.24 to 1.04)
	Superoinferior	−0.33 (−0.58 to −0.08)	0.46 (0.21 to 0.71)	Yaw	−0.22 (−0.34 to −0.10)	0.17 (0.05 to 0.29)
Mandible	Mediolateral	−3.21 (−5.63 to −0.79)	4.56 (2.14 to 6.98)	Pitch	−2.17 (−3.50 to −0.84)	2.09 (0.77 to 3.42)
	Anteroposterior	−1.82 (−3.57 to −0.07)	3.81 (2.06 to 5.56)	Roll	−1.16 (−1.95 to −0.37)	1.38 (0.59 to 2.18)
	Superoinferior	−1.42 (−2.73 to −0.10)	2.80 (1.49 to 4.11)	Yaw	−1.35 (−2.54 to −0.15)	2.49 (1.30 to 3.69)
Maxilla	Mediolateral	−2.67 (−4.59 to −0.75)	3.50 (1.58 to 5.42)	Pitch	−5.46 (−9.81 to −1.12)	8.49 (4.15 to 12.84)
	Anteroposterior	−1.84 (−3.90 to 0.22)	4.77 (2.71to 6.83)	Roll	−2.76 (−5.09 to −0.44)	4.72 (2.39 to 7.04)
	Superoinferior	−1.55 (−2.58 to −0.51)	1.78 (0.74 to 2.81)	Yaw	−1.92 (−3.11 to −0.72)	1.92 (0.73 to 3.12)

Compared to this overall accuracy, the translational and rotational differences due only to the splint were lower and were all within the accuracy cutoff (Table 3). Nevertheless, the width of the limits of agreement (range between lower and upper limits) showed that rotational differences could be particularly large (Table 4).

**Table 3:** Splint accuracy (*Root Mean Square Deviation*) of 3D translational and rotational differences between the planned and post-operative images

Translational difference		Rotational difference	
Mediolateral	1.18	Pitch	1.03
Anteroposterior	1.63	Roll	0.63
Superoinferior	1.03	Yaw	1.09

**Table 4:** Splint accuracy (*Bland-Altman upper and lower limits*) of 3D translational and rotational differences between the planned and post-operative images

Translational difference (95 % CI)			Rotational difference (95 % CI)		
	Lower limit	Upper limit		Lower limit	Upper limit
Mediolateral	−2.71 (−4.12 to −1.30)	1.83 (0.41 to 3.24)	Pitch	−9.33 (−14.16 to −4.49)	6.22 (1.38 to 11.06)
Anteroposterior	−2.23 (−4.09 to −0.37)	3.74 (1.88 to 5.60)	Roll	−4.60 (−6.92 to −2.27)	2.87 (0.54 to 5.19)
Superoinferior	−1.63 (−2.87 to −0.39)	2.35 (1.11 to 3.59)	Yaw	−1.51 (−2.81 to −0.22)	2.65 (1.35 to 3.94)

The translational difference due to screw deformation was higher for the mandible than for the skull or the maxilla and represented a relatively large source of error since it varied from 25.5 % (medio-lateral axis of the mandible: 0.51/2.00) to 66.9 % (antero-posterior axis of the mandible, 1.13/1.69) (Table 5).

**Table 5:** RMSD to estimate inaccuracy due to screw deformation

	Distance	
Skull	Mediolateral	0.14
	Anteroposterior	0.22
	Superoinferior	0.18
Mandible	Mediolateral	0.51
	Anteroposterior	1.13
	Superoinferior	0.40
Maxilla	Mediolateral	0.24
	Anteroposterior	0.17
	Superoinferior	0.20

## Discussion

The aim of this cadaveric study was to evaluate the accuracy of computer-assisted design and manufacturing (CAD/CAM) generated splints used for maxillary repositioning during a Le Fort I osteotomy. Our results provide the following considerations. First, the accuracy related to the imaging process evaluated by the differences between pre- and post-operative translational and rotational movements as measured on the only structure that was not repositioned during the surgery such as the skull was found to be excellent. The calculation of the accuracy was directly influenced by: a) the imaging acquisition error related to the multi-slice CT scan used (CT-Sensation 64 = within 0.3 mm); b) the intrinsic software error related to the procedure of 3D segmentation and fusion between planned pre-operative and post-operative CT scan images. This step was made by powerful algorithms that allowed for a very rapid automated calculation. The precision rate of the surgical planning software used in the present study as given by the manufacturers was within 1 mm; c) the technical error in determining the position of the screws within the CT images for measurement calculation; d) the human errors that may potentially occur at every step of either the computer planning or the surgical procedure cannot be ignored although it is very difficult to quantify them; e) the whole procedure of the accuracy assessment itself. Second, maxillary repositioning was found to be accurate according to the standard permitted by several researchers who have set the accuracy cutoff for translational movements at 2 mm considering that differences that are not larger than 2 mm may not likely be noticeable to the naked eye or even be perceived by patients and 4° for the rotational movements of the occlusal plane [16–18]. Conversely, other investigated have reported the accuracy between the actual and planned facial landmark measurements permitted for clinical use to be within 0.5 mm [19]. To the best of our knowledge, there is no consensus on the tolerable margin of error for a specific technique to be considered accurate. Moreover, it should also be pointed out that using mean difference resulted in an overly optimistic accuracy assessment since positive and negative differences cancel each other. In fact, when using the Bland & Altman method to establish the lower and upper limits of accuracy with 95 % confidence intervals around these limits, the results showed greater variability and thus showcased a lower overall accuracy. Thus, the differences measured for the maxilla quantify the error related to the surgical procedure as well as the error related to the splints. No doubt this was the most important information, since this is finally what is obtained when applying such a surgical procedure onto the patient. The calculation of this accuracy was determined by: a) the difference of the condylar repositioning in the centric relation between the planned and the actual post-operative images; b) the error of the 3D optical scans used for registering the dental models; c) the error of the registration process of the digital models after 3D optical scanning within the CT images; and d) the error of the manufacturing process of the splint.

Third, the main part of the differences between the post-operative and planned position of the maxilla was due to a difference in mandibular position and not due to inaccuracies of the splint itself (e.g., splint design, fit of splint onto teeth, positioning of the splint onto teeth). In fact, by calculating the difference between the maxillary and mandibular deviations, we obtained the true error related to the splint, which was less than 2 mm, thus confirming an acceptable accuracy of the digital splints. These results were confirmed by a re-analysis matching post-operative onto pre-operative planning scan images of the mandible and not onto the pre-operative planning scan images of the skull. By doing so, the deviations between planned and post-operative images of the maxilla were only related to the splint itself and thus to the above-mentioned sources of errors related to the manufacturing process. In fact, with this analysis, the surgical errors due to the difference in condylar positioning between planning and surgery could be excluded and were thus not taken into account in the final calculation.

The CAD/CAM splints have been described in the literature as the most accurate and reliable method for orthognathic treatment, especially for asymmetrical cases [6–16]. Previous clinical studies on the CAD/CAM splints have highlighted several factors that could potentially have a non negligible impact on the overall accuracy measurement. These include the osteosynthesis procedure that may influence the final position of the maxilla, the image’s metal artifacts related to the plates, which can cause aberrant values that are difficult to take into account during the registration and fusion process, the errors related to the virtual mandibular autorotation necessary to obtain a centric relation in cases where the postoperative CT-scan has been taken with the patient’s mouth open, the timing of post-operative imaging that could also influence the results as the bone segments may suffer some slight displacements and remodeling under muscular loading [16]. In our study, the mobilized maxillo-mandibular complex was locked into the splint and secured to the skull base with non-metallic bone wiring and the post-operative CT scan was taken a few days after the surgery.

Hsu et al. concluded that a combination of the computer-aided surgical simulation and the CAD/CAM splint resulted in excellent positional and orientation accuracy for the maxilla and mandible and excellent accuracy for the maxillary dental-midline position [16]. In this multicenter clinical study, the authors measured linear and angular deviations between the centroids of mobilized bone segments using dental landmarks. Similar to our study, the authors reported large differences (>4 mm) between planned and actual outcomes in some cases. The authors stated that this was due to failure to capture centric relation of the mandibular condyle. Our results were similar and showed that CAD/CAM splints were reliable for replicating the 3D virtually planned maxillo-mandibular relation and that the error related to the surgical mandibular repositioning was predominant [16]. As long as the maxillary repositioning remains rigidly tied to the mandible via a splint, the maxillary repositioning’s accuracy will always be dependent on the mandibular repositioning during surgery. For this reason, the unsolved difficulty of reproducing the planned centric relation of the condyles has always played a major role in limiting the potential benefit of 3D-virtual planning and CAD-CAM splints.

The present study has demonstrated that digital CAD/CAM splints resulted in acceptable accuracy with respect to the capacity of reproducing the planned maxillo-mandibular repositioning. However the inaccuracy in the maxilla-mandibular repositioning was mainly related to the difference in the condylar post-operative repositioning compared to the pre-operative position and negligibly to the splint itself.
